# Health service use and health outcomes among international migrant workers compared with non-migrant workers: A systematic review and meta-analysis

**DOI:** 10.1371/journal.pone.0252651

**Published:** 2021-06-09

**Authors:** Frank Pega, Srinivasan Govindaraj, Nguyen Toan Tran

**Affiliations:** 1 Environment, Climate Change and Health Department, World Health Organization, Geneva, Switzerland; 2 Australian Centre for Public and Population Health Research, Faculty of Health, University of Technology, Sydney, NSW, Australia; 3 Faculty of Medicine, University of Geneva, Geneva, Switzerland; Universita degli Studi di Firenze, ITALY

## Abstract

**Objectives:**

The review aimed to synthesise recent evidence on health service use and health outcomes among international migrant workers, compared with non-migrant workers.

**Methods:**

A search was carried out in MEDLINE, PubMed, Embase, and CINAHL for studies published between Jan 1, 2010, and Feb 29, 2020. Included outcomes were: occupational health service use, fatal occupational injury, HIV, and depression. Two authors independently screened records, extracted data, assessed risk of bias and judged quality of evidence. We meta-analysed estimates and conducted subgroup analyses by sex, geographical origin, geographical destination, and regularity of migration.

**Results:**

Twenty-one studies were included comprising >17 million participants in 16 countries. Most studies investigated regular migrant workers in high-income destination countries. Compared with non-migrant workers, migrant workers were less likely to use health services (relative risk 0·55, 95% confidence interval 0·41 to 0·73, 4 studies, 3,804,131 participants, I^2^ 100%, low quality of evidence). They more commonly had occupational injuries (1·27, 95% confidence interval 1·11 to 1·45, 7 studies, 17,100,626 participants, I^2^ 96%, low quality of evidence). Relative risks differed by geographical origin and/or destination. There is uncertainty (very low quality of evidence) about occupational health service use (0 studies), fatal occupational injuries (5 studies, N = 14,210,820), HIV (3 studies, N = 13,775), and depression (2 studies, N = 7,512).

**Conclusions:**

Migrant workers may be less likely than non-migrant workers to use health services and more likely to have occupational injuries. More research is required on migrant workers from and in low- and middle-income countries, across migration stages, migrating irregularly, and in the informal economy.

## Background

An *international migrant worker* is as “a person who migrates or has migrated to a country of which they are not a national with a view to being employed other than as an own-account worker” (p. 3) [1]. Globally, 164 million people are international migrant workers; 41 6% are females [2]. Internal (in-country) migrant workers are a different population.

International migrant workers (hereafter “migrant workers”) may face unique work-related and occupational safety and health challenges. A recent systematic review and meta-analysis found that 22% (95% confidence interval (CI) 7–37) of migrant workers had occupational injuries and 47% (95% CI 29–64) had psychiatric and physical morbidities [3]. Their health service use and health outcomes may depend on their sex; their migration stage (i.e., pre-departure, travel, interception, destination, and return) [4]; region (or country) of destination or origin, or both; whether they migrated regularly or irregularly; and whether they work in the formal or informal economy, amongst other factors.

The health service use and health outcomes may differ between migrant and non-migrant workers, but previous systematic review evidence on this topic is scarce. The latest comprehensive systematic review of such evidence was published in 2007, covered the years 1990 to 2005, included 48 qualitative and quantitative studies, and found migrant workers had higher risks of fatal and non-fatal occupational injuries than non-migrant workers [5]. A 2013 systematic review of 19 qualitative and quantitative studies on fatal and non-fatal occupational injuries in China concluded that these injuries were more prevalent among (internal) migrant workers than non-migrant workers [6]. A 2017 systematic found four studies on fatal and non-fatal occupational injuries among migrant workers from Nepal compared with non-migrant workers and concluded this evidence is very uncertain [7]. A 2018 systematic review of 82 studies published between 2000 and 2016 on differences by migrant status in working conditions and occupational health outcomes in Canada and Europe found that migrant workers may experience relatively poorer working conditions and occupational health [8]. However, it noted uncertainty from large data gaps, heterogeneous study populations, and too few prospective cohort studies [8]. No meta-analysis has been published to-date.

Policy-makers require comprehensive, up-to-date systematic review and meta-analytic evidence on these differences to design, plan, cost, implement and evaluate laws, policies, and interventions that promote the health and wellbeing of workers. The last comprehensive systematic review on the topic covered data from over a decade ago, and research on migration, work and health has accelerated since. A new systematic review and first meta-analyses (if feasible) are warranted. We aimed to systematically review and meta-analyse recent evidence from quantitative studies on health service use and health outcomes among migrant workers, compared with non-migrant workers, published over the past decade (2010–2020).

## Methods

### Protocol

Before commencing the systematic review, we developed a protocol that guided all aspects of the systematic review.

#### Search strategy

We searched MEDLINE, PubMed, Embase, CINAHL and OpenGrey in March 2020 for study records published between Jan 1, 2010, and Feb 29, 2020. The search strategy for MEDLINE was adapted to search other databases (see S1 Appendix). We hand searched reference lists of previous systematic reviews [5–7] and records of studies included in this systematic review. The first 100 hits on Google and GoogleScholar and the webpages of ILO, IOM, UNHCR, and WHO were also searched. Experts were asked to identify eligible published and unpublished studies.

#### Eligibility criteria

The outcomes of interest were any health service use, any occupational safety and health service use, death from an occupational injury, any non-fatal occupational injury, HIV infection and clinical depression. S1 Table presents the eligibility criteria for populations, comparators, and outcomes. All included outcomes are or align with Sustainable Development Goals indicators [9] with relevance for health and migration (S2 Table).

#### Study selection

At least two review authors independently screened the titles and abstracts of potentially eligible study records from the search results against the eligibility criteria. Of records that we identified as potentially eligible, review authors independently screened the full texts to determine inclusion in the systematic review. The third review author resolved any disagreements.

#### Data collection process

Data extraction was conducted independently by two review authors. A standard data extraction sheet was developed and trialled by the data extractors. From each included study, we extracted data on study design (data analytic method, model used, and confounder adjustment), participants (number and type of participants), comparator, outcomes, relative risk (RR) measure, point estimate and 95% CI. The third review author resolved disagreements.

#### Risk of bias assessment

Following Cochrane’s approach, we assessed risk of bias by outcome at the level of the individual study and then at the level of the entire body of evidence. Risk of bias assessment tools are lacking for studies of *differences* in prevalence. We used the RoB-SPEO tool [10] for studies estimating prevalence in occupational health. The assessed domains were: (1) bias in selection of participants into the study; (2) bias due to a lack of blinding of study personnel; (3) bias due to exposure misclassification; (4) bias due to incomplete exposure data; (5) bias due to conflict of interest; (6) bias due to selective reporting of exposures; (7) bias due to difference in numerator and denominator; and (8) other bias. For each domain, the risk of bias was rated as “high”, “low” or “unclear” [11]. Two review authors conducted the assessment independently, and the third author resolved disagreements. Consensus ratings for each domain for each study are presented in a “Summary of risk of bias” table [11].

#### Evidence synthesis

Two authors independently assessed the clinical heterogeneity of included studies on the same outcome, with the third author resolving differing opinions. We combined studies judged sufficiently homogenous empirically in a meta-analysis. Measures of relative differences other than RRs (eg, odds ratios) were converted into RRs if possible using Cochrane’s guidance [12]. Studies were pooled using the inverse variance method with random effects models, because included studies estimated different, yet related effects [13]. Review Manager (RevMan 5.3) computer software was used [14]. We assessed statistical heterogeneity of studies with the I^2^ statistic. We expected high levels of heterogeneity, so report pooled effect estimates from meta-analyses also when statistical heterogeneity is high (e.g. I^2^ > 95%). For outcomes with evidence rated as “very low quality” (meaning that we were very uncertain about the outcome), we report forest plots of meta-analyses, but do not report pooled estimates. The results of the evidence synthesis are presented in a “Summary of findings” table [15].

### Additional analyses

Subgroup analyses were conducted by sex; WHO region of destination, WHO region of origin, and regularity of the migration. Such analyses were only conducted of data presented in main meta-analyses with pooled estimates.

### Quality of evidence assessment

For each outcome, we assessed the quality of its entire body of evidence, using the Grading of Recommendations Assessment, Development and Evaluation (GRADE) approach [16]. Our assessment considered risk of bias, inconsistency, indirectness, imprecision, and size of the RR estimates. We applied the ratings “high”, “moderate”, “low” and “very low”. Our assessment started at “high”. For each domain, we downgraded by one level for serious concerns and by two levels for very serious concerns. Evidence was upgraded by one level and two levels if the estimated RRs were high (≥ 2.5) and very high (≥ 5.0), respectively. The “Summary of findings” table includes quality of evidence ratings and their justification [15].

## Results

### Study selection

**Fig 1:**
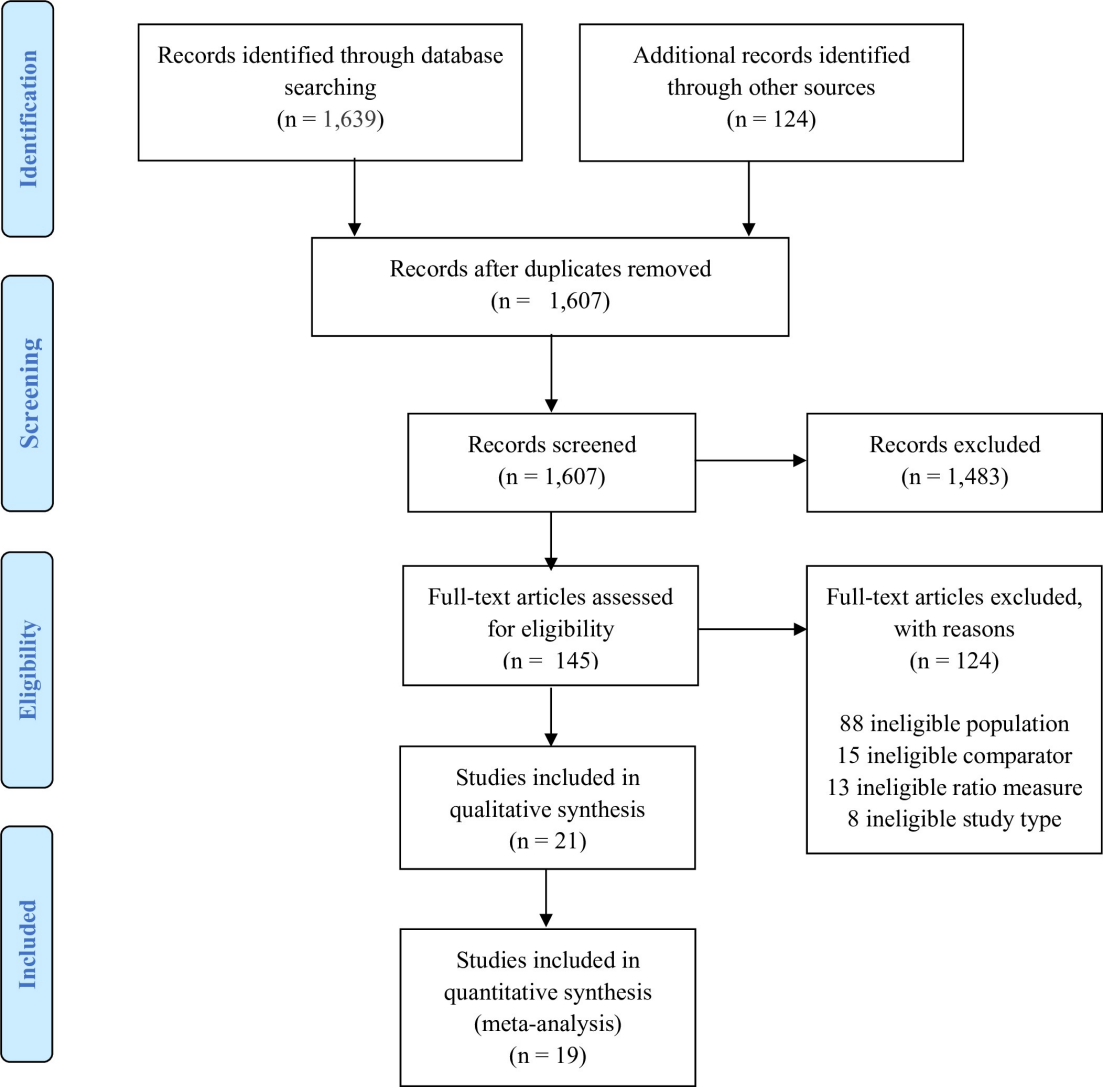
Flow diagram of study selection.

Of 1607 study records identified by our search, 21 studies with 22 study records fulfilled the inclusion criteria and were included in this review (Fig 1) [17–38].

### Study characteristics

The included studies comprised >17 million participants in 16 countries within five WHO regions (Africa, Americas, Eastern Mediterranean, Europe, and Western Pacific) (Table 1). Thirteen included studies were cohort studies, and eight were cross-sectional. Most studies investigated regular migrant workers from high-income countries working in the formal economy of high-income destination countries.

**Table 1:** Characteristics of included studies.

Study	Study location	Migrant workers	Non-migrant workers	Study period	Occupation or industrial sector	Type of study	Number of participants or events	Female	Age (in years)	Outcomes
Al-Thani 2015 [17]	One site, Qatar	Nationals of Bangladesh, Egypt, India, Nepal, Philippines and Sri Lanka	Nationals of Qatar	4 years (2010–13)	Labourers	Cohort study	2,015 participants	0·02	32 (mean)	Has died from an occupational injury, Has had any occupational injury (major injury only), measured with the Abbreviated Injury Score
Biering 2017 [18, 19]	One site, Denmark	Nationals of old EU, new EU and other countries	Nationals of Denmark	11 years (2003–13)	All workers	Cohort study	63,601 participants	0·47	Unclear	Has had any occupational injury, measured with physician or employer report
Byler 2018 [20]	United States of America (USA)	Workers born in Africa, Americas (other than the USA), Asia, Europe and other regions	Workers born in the United States of America	8 years (2003–10)	All workers	Cohort study	Unclear number of participants (39,048 observations)	0·08	16–67 (range)	Has died from an occupational injury, measured with administrative records
Cha 2014 [21]	Republic of Korea	Nationals of Bangladesh, Egypt, India, Nepal, Philippines and Sri Lanka	Nationals of the Republic of Korea	3 years (2005–07)	All workers	Cohort study	341,359 + 1,252,8879 participants	0·17	Any age (range)	Has died from an occupational injury, Has had any occupational injury (non-fatal only)
Dias 2017 [22]	Unclear number of sites, Portugal	Nationals of countries in the Africa, Americas, and Europe (other than Portugal)	Nationals of Portugal		Sex workers	Cross-sectional study	853 participants	1·0	35·9 (mean)	Has used any health service; measured with self-reported lifetime use of HIV testing
Giraudo 2017 [23]	Italy	Nationals of countries in Africa, Asia (except Japan and Republic of Korea), Latin America, and Central and Eastern Europe	Nationals of Italy	6 years (2000–05)	Manufacturing, Construction, and Services (industrial sectors)	Cohort study	397,986 workers with 6,629 events	0·00	16–55 (range)	Has had any occupational injury (serious occupational injury resulting in four or more days of absence)
Goldenberg 2014 [24]	Vancouver, Canada	Born outside of Canada	Born in Canada	3 years (2010–12)	Sex workers	Cross-sectional study	650 participants	1·00	34 (median)	Has human immune virus, measured with laboratory tests
Ismayilova 2014 [25]	Almaty, Kazakhstan	Nationals of Azerbaijan, China, Kyrgyzstan, Russia, Tajikistan, Turkey and Uzbekistan	Nationals of Khazakhstan (internal migrants only)	4 months (2007)	Market workers	Cross-sectional study	450 participants	0·50	27·7 (mean)	Has had clinical depression in last week, measured with the Depression Subscale of the Brief Symptom Inventory
López-Arquillos 2016 [26]	Spain	Nationals of a country other than Spain	Nationals of Spain	6 years (2003–08)	Automotive repair workshop workers	Cohort study	89,954 events	0·03	Unclear	Has had any occupational injury (bone fracture only)
Mc Grath-Lone 2014 [27]	England, United Kingdom	Born outside of the United Kingdom	Born in United Kingdom	1 year (2011)	Sex workers	Cohort study	2,704 participants	1·00	28 (migrant), 29 (non-migrant) (mean)	Has human immunodeficiency virus, measured with a medical test
Rakprasit 2017 [28]	Thailand	Nationals of countries other than Thailand	Nationals of Thailand	1 year (2011)	All workers	Cohort study	803,817 participants	0·59	18–59 (range)	Has used any health services (for diarrhoea only), measured with administrative records
Reid 2016 [29]	Australia	Born in a country other than Australia	Born in Australian	12 years (1991–2002)	All workers	Cohort study	5,156 events	0·50	15–64 (range)	Has died from an occupational injury
Ricco 2019 [30]	Autonomous Province of Trento, Italy	Born in a country in the Eastern Mediterranean	Born in Italy	14 years (2000–13)	All workers	Cohort study	Unclear	Unclear	Unclear	Has had any occupational injury, measured with administrative records
Richter 2014 [31]	Three cities, South Africa	Nationals of Botswana, Democratic Republic of the Congo, Eswatini, Malawi, Mozambique, Namibia, Nigeria, Zambia, and Zimbabwe	Nationals of South Africa	1 year (2010)	Sex workers	Cross-sectional study	1,653 participants	1·00	29.7 (mean)	Has used any health service in last month
Rubiales-Gutierrez 2010 [32]	Spain	Nationals of countries with a low Human Development Index score in Asia, Europe, Latin America and Oceania	Nationals of Spain	1 year (2008)	All workers	Cross-sectional study	10,927 participants	0·42 (migrant), 0·41 (non-migrant)	Unclear	Has had any occupational injury
Salvatore 2013 [33]	Italy	Nationals of high migration pressure countries^a^	Nationals of Italy	1 year (2007)	All workers	Cohort study	60,528 participants	0·62 (migrant), 0·62 (non-migrant)	Unclear	Has had any occupational injury
Sieberer 2012 [34]	Germany	Nationals of Kazakhstan, Poland, Russia, Turkey and other countries	Nationals of Germany	2 months (2008)	Health workers	Cross-sectional study	2,796 participants	0·74	≥18 (range)	Is clinically depressed (CESD score ≥ 23), measured with the Center of Epidemiological Studies Depression Scale
Steege 2014 [35]	USA	Born outside the USA and USA territories	Born in the USA	5 years (2005–09)	All workers	Cohort study	27,000 events	Unclear	≥15 (range)	Died from an occupational injury,
Straiton 2014 [36]	Norway	Born in countries outside of Norway, including Germany, Iraq, Pakistan, Poland and Sweden	Born in Norway	1 year (2008)	All workers	Cross-sectional study	2,962,408 individuals	0·44	38.5 (mean)	Has used any health service (psychological diagnosis from general practitioner only)
Wong 2011 [37]	One site, Hong Kong, China	Country of origin other than China	Country of origin China	3 years (2005–07)	Sex workers	Cohort study	503 participants	1·00	Unclear	Has human immune virus, measured with laboratory test
Zhou 2013 [38]	35 sites, Guangxi, China	Nationals of Viet Nam	National of China	1 year (2010)	Sex workers	Cross-sectional study	12,622 participants	1·00	Mainly 20–39 (range)	Has human immune virus, measured with laboratory test

^a^ High migration pressure countries do not include in Europe, Austria, Belgium, Denmark, Finland, France, Germany, Greece, Ireland, Luxemburg, Netherlands, Portugal, United Kingdom, Spain, Sweden, Andorra, Cyprus, Iceland, Liechtenstein, Malta, Monaco, Norway, San Marino, Switzerland, and Vatican City; in North America, Canada and the United States; in Asia, Israel and Japan; and all of Oceania.

### Risk of bias

For each outcome, we judged the risk of bias of the body of evidence to be high (see S1 and S2 Figs). Over half of all included studies (13 of 21 studies) were judged to carry a high risk of selection bias because they analysed study samples that were non-representative of national populations of either or both of migrant workers and non-migrant workers. Of note, most studies captured neither migrant workers from irregular migration nor workers in the informal economy. The risk of performance and detection bias was often judged to be overall low because most studies relied on administrative data collected for purposes other than comparing the health of migrant workers with that of non-migrant workers. Most studies carried a high risk of bias from selective reporting because migrant workers may have underreported adverse outcomes at disproportionately higher rates due to relatively higher fear of losing their work if reporting adverse outcomes. Risks of confounding and of bias from conflict of interest were generally low. Several studies carried a risk of bias from differences between the numerator and the denominator.

### Findings

#### Use of any health services

**Fig 2:**
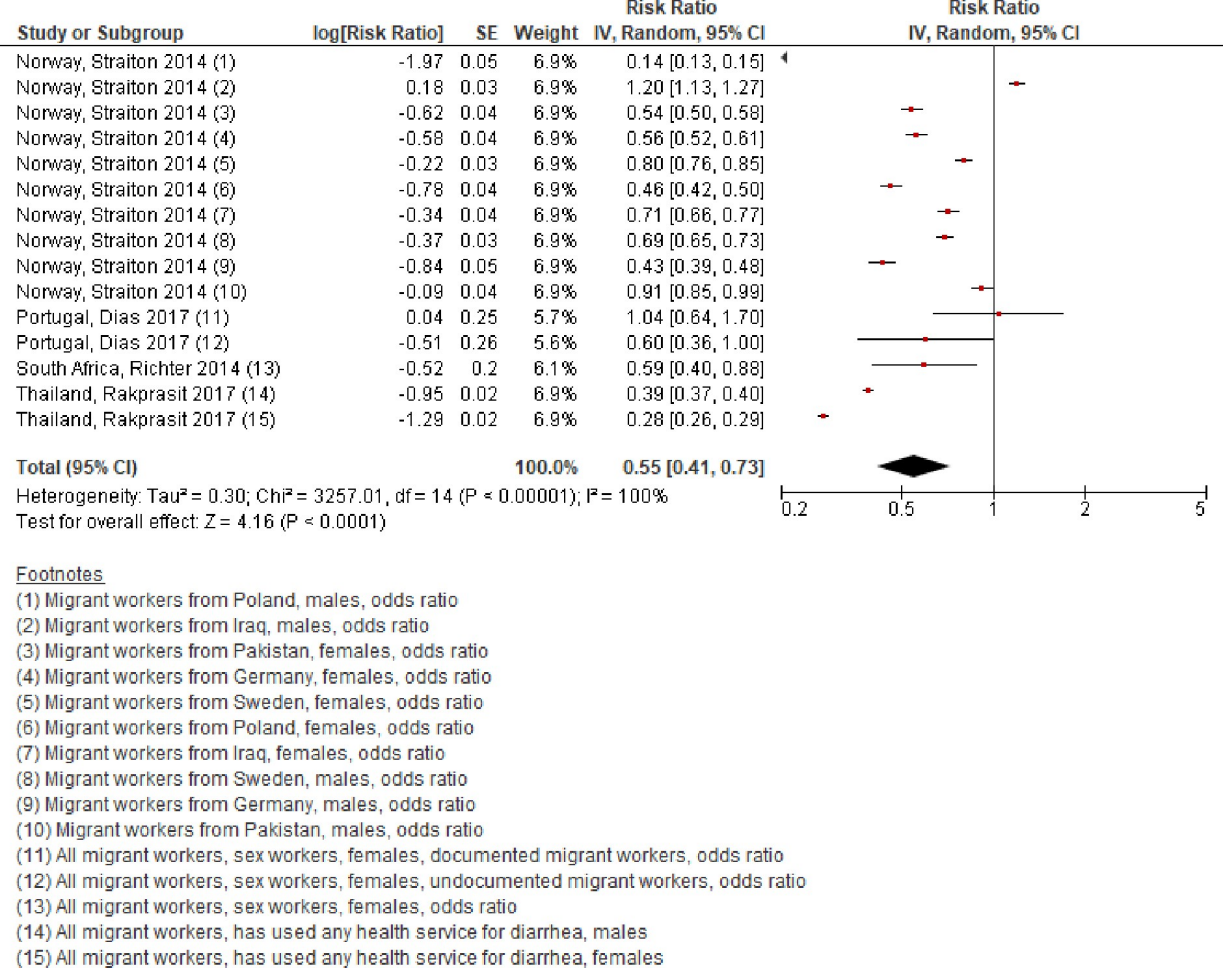
Has used any health services, migrant workers compared with non-migrant workers, 2010–20.

Four studies with a total of 3,804,131 participants from four countries in three WHO regions (Africa, Europe, and South-East Asia) reported an estimate of the use of any health service among migrant workers, as compared with non-migrant workers [22, 28, 31, 36]. The populations and definitions and measurements for this outcome were somewhat heterogeneous (Table 2). Nevertheless, we judged them to be sufficiently comparable to combine them in one meta-analysis. Compared with non-migrant workers, migrant workers were an estimated 45% less likely to use any health services (RR 0·55, 95% CI 0·41 to 0·73, I^2^ 100%, Fig 2).

**Table 2:** Subgroup analyses for outcomes with meta-analysis.

Subgroup	Outcome	
	Has used any health service (4 studies included in meta-analysis) [22, 28, 31, 36]	Has had any occupational injury (7 studies included in meta-analysis) [17, 18, 21, 23, 30, 32, 33]
Sex	(4 studies) [22, 28, 31, 36]	(1 study) [32]
Females	0·58 (0·41 to 0·81)	1·66 (1·21 to 2·28)
Males	0·51 (0·30 to 0·88)	1·19 (0·93 to 1·52)
Test for subgroup differences	P = 0·70, I^2^ = 0%	P = 0·12, I^2^ = 59%
Country of destination (WHO region)	(4 studies) [22, 28, 31, 36]	(7 studies) [17, 18, 21, 23, 30, 32, 33]
Africa	0·59 (0·40 to 0·87)	-
Americas	-	-
Eastern Mediterranean	-	4·76 (1·75 to 12·93)
Europe	0·60 (0·44 to 0·80)	1·22 (1·09 to 1·36)
South-East Asia	0·33 (0·23 to 0·46)	-
Western Pacific	-	1·75 (1·68 to 1·82)
Test for subgroup differences	P = 0·02, I^2^ = 76%	P < 0·01, I^2^ = 95%
Country of origin (WHO region)	(2 studies) [31, 36]	(3 studies) [17, 18, 30]
Africa	0·59 (0·40 to 0·87)	-
Americas	-	-
Eastern Mediterranean	0·80 (0·57 to 1·15)	1·13 (1·04 to 1·22)
Europe	0·45 (0·29 to 0·71)	1·01 (0·81 to 1·25)
South-East Asia	-	4·76 (1·75 to 12·93)
Western Pacific	-	-
Test for subgroup differences	P = 0·13, I^2^ = 50%	P = 0·01, I^2^ = 78%
Regularity of migration	(1 study) [22]	(0 studies)
Irregular	0·61 (0·37 to 0·99)	-
Regular	1·04 (0·64 to 1·70)	-
Test for subgroup differences	P = 0·13, I^2^ = 57%	-

We downgraded the quality of this body of evidence by two grades from “high” to “low” quality of evidence. We downgraded by one grade each for serious risk of bias and serious indirectness. We did not downgrade for inconsistency; we had expected heterogeneity to be very high, even due to the heterogeneous study population alone, and we also found very high heterogeneity in the analysis. In conclusion, migrant workers may be less likely to use any health service than non-migrant workers. Further research is very likely to have an important impact on our confidence in the conclusion and is likely to change it.

In subgroup analyses by WHO region (Table 2), the likelihood among migrant workers was reduced in all WHO regions with data included in the systematic review, but it may be particularly lower in South-East Asia. There may be considerable differences in this outcome between regions (Test for subgroup differences: p = 0·02).

#### Use of any occupational safety and health services

Our systematic review identified no eligible study on the relative likelihood of using any occupational safety and health service among migrant workers, compared with non-migrant workers.

#### Fatal occupational injury

**Fig 3:**
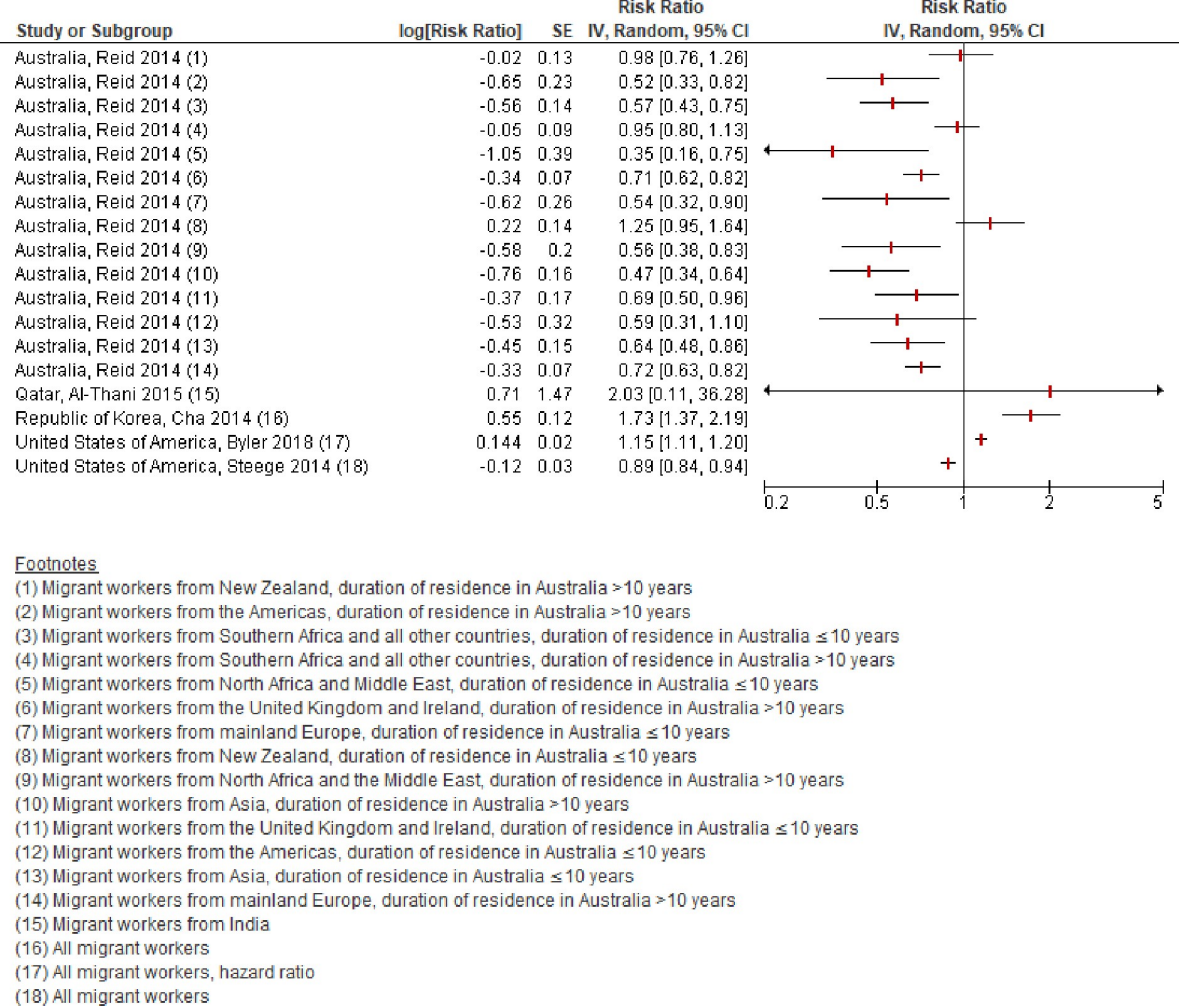
Has died of an occupational injury, migrant workers compared with non-migrant workers, 2010–20.

Five studies with a total of 14,210,820 participants reported an estimate of having a fatal occupational injury among migrant workers, compared with among non-migrant workers [17, 20, 21, 29, 35]. We judged the studies to potentially be sufficiently homogenous and combined them in a meta-analysis (Fig 3). We downgraded the quality of this body of evidence by three grades from “high” to “very low” quality of evidence. We downgraded by two grades for very serious risk of bias and by one grade for serious indirectness. Because of the very low quality of evidence, we do not present a pooled estimate in the forest plot. We are very uncertain about this outcome among migrant workers, compared with non-migrant workers.

#### Any occupational injury

**Fig 4:**
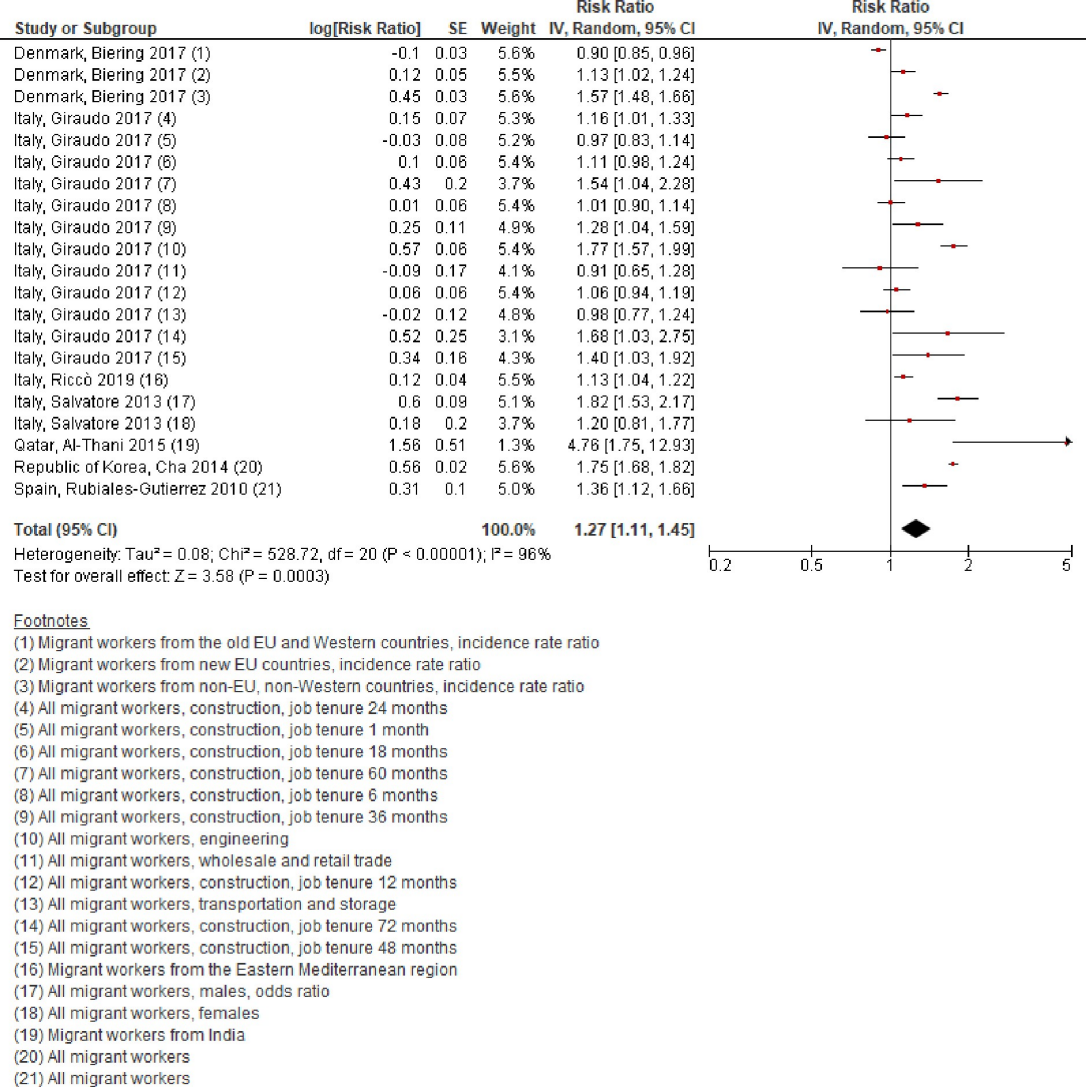
Has had any occupational injury, migrant workers compared with non-migrant workers, 2010–20.

Eight studies with a total of 17,100,626 participants from five countries in three WHO regions (Eastern Mediterranean, Europe, and Western Pacific) reported an estimate of having had any occupational injury among migrant workers, compared with non-migrant workers [17, 18, 21, 23, 26, 30, 32, 33]. We judged seven studies [17, 18, 21, 23, 30, 32, 33] with 21 individual estimates from 13,063,936 participants to potentially be sufficiently homogenous despite some heterogeneity in the population and in the outcome definition and measurement. Migrant workers were 27% more likely than non-migrant workers to have an occupational injury (RR 1·27, 95% CI 1·11 to 1·44, I^2^ 96%) (Fig 4). The eighth study [26] reported an OR of 1·07 without an estimate of statistical variance for the prioritised measure (occupational bone fracture) and was therefore not be included in the meta-analysis; however, we carried out a sensitivity analysis, where we added this study [26] using the median standard error across the included individual effect estimates from the other included studies (0.08). The pooled effect estimate was almost identical, with an OR of 1.26 (95% CI 1.11 to 1.43; see S3 Fig for forest plot).

We downgraded this body of evidence by two grades to “low quality evidence” for serious risk of bias and serious indirectness. In conclusion, migrant workers may perhaps have a higher likelihood of having any occupational injury than non-migrant workers. Further research is very likely to have an important impact on our confidence in the conclusion and is likely to change it.

Subgroup analyses by WHO region found that the risk among migrant workers was elevated in all WHO regions with data included in this systematic review, but it may be particularly elevated in the Eastern Mediterranean and Western Pacific (Table 2). There may be considerable differences in this outcome between regions (Test for subgroup differences: p < 0·000).

#### HIV

Four studies with a total of 13,775 participants from three countries in three WHO regions (Americas, Europe, and Western Pacific) reported an estimate of having HIV among migrant workers, compared with among non-migrant workers [24, 27, 37, 38]. All studies investigated differences among sex workers. We judged three studies [24, 37, 38] to potentially be sufficiently homogenous and combined them in a meta-analysis (see S4 Fig). The fourth study [27] reported that a ratio of the odds of migrant workers divided by the odds of non-migrant workers could not be calculated because no non-migrant workers had HIV. We downgraded the quality of this body of evidence by three grades for serious risk of bias, indirectness and imprecision from “high” to “very low” quality of evidence. Because of the very low quality of evidence, we do not present a pooled estimate in the forest plot. We are very uncertain about this outcome among migrant workers, compared with non-migrant workers.

#### Depression

Two studies with a total of 7,512 participants from two countries in Europe reported an estimate of being clinically depressed among migrant workers, compared with non-migrant workers [25, 34]. We judged both studies to potentially be sufficiently homogenous and combined them in a meta-analysis (see S5 Fig). We downgraded the quality of this body of evidence by three grades for serious risk of bias, indirectness and imprecision to “very low” quality of evidence and do not present a pooled estimate in the forest plot. We are very uncertain about this outcome.

## Discussion

This systematic review and meta-analysis included 21 studies of >17 million participants in 16 countries in five regions [17–38]. Most studies investigated regular migrant workers in high-income destination countries. Compared with non-migrant workers, migrant workers may be less likely to use any health service (Table 3 summarises the findings). They may be more likely than non-migrant workers to have an occupational injury. In subgroup analyses, RRs of these outcomes differed considerably between geographical origin and/or destination regions, but not by sex. We are very uncertain about the likelihood of migrant workers, compared with non-migrant workers, to have used any occupational health services; died from an occupational injury; HIV; and depression.

**Table 3:** Summary of findings: Use of health services and health outcomes among migrant workers compared with non-migrant workers.

Population: Migrant workers in country of destination						
Setting: High- and upper middle-income countries						
Comparator: Non-migrant workers in the same country						
Outcome	Illustrative comparative risks* (95% CI)		Relative effect (95% CI)	No of participants or events (studies)	Quality of evidence	Comments
	Assumed risk	Corresponding risk				
Has used any health services	The assumed risk in non-migrant workers is **60 per 100**	The corresponding risk in migrant workers is **33 per 100** (25 to 45)	RR 0·55 (0·41 to 0·73)	3,804,131 participants (4 studies)	⊕⊕⊝⊝	Better outcomes for migrant workers indicated by higher values. Migrant workers may be less likely to use any health services than non-migrant workers.
					Low^a^^,^^b^	
Has used any occupational safety and health services	-	-	-	-	-	No evidence available on this outcome
Has died from any occupational injury	-	-	-	14,210,820 participants and 130,774 events (4 studies)	⊕⊝⊝⊝	Better outcomes for migrant workers indicated by lower values. We are very uncertain about this outcome among migrant workers, compared with non-migrant workers.
					Very low^a^^,^^c^	
Has had any occupational injury	The assumed risk in non-migrant workers is **34 per 10,000**	The corresponding risk in migrant workers is **43 per 10,000** (38 to 49)	RR 1·27 (1·11 to 1·44)	17,100,626 participants (11 studies)	⊕⊕⊝⊝	Better outcomes for migrant workers indicated by lower values. Migrant workers may be more likely to have any occupational injury than non-migrant workers.
					Low^a^^,^^b^	
Has human immunodeficiency virus				13,775 participants (4 studies)	⊕⊝⊝⊝	Better outcomes for migrant workers indicated by lower values. We are very uncertain about this outcome among migrant workers, compared with non-migrant workers.
					Very low^a^^,^^b^^,^^d^	
Has clinical depression				7,512 participants (2 studies)	⊕⊝⊝⊝	Better outcomes for migrant workers indicated by lower values. We are very uncertain about this outcome among migrant workers, compared with non-migrant workers.
					Very low^a^^,^^b^^,^^d^	
**High quality**: further research is very unlikely to change our confidence in the estimate of effect.						
**Moderate quality**: further research is likely to have an important impact on our confidence in the estimate of effect and may change the estimate.						
**Low quality**: further research is very likely to have an important impact on our confidence in the estimate of effect and is likely to change the estimate.						
**Very low quality**: we are very uncertain about the estimate.						

* The basis for the assumed risk is the median control group risk across studies. The corresponding risk (and its 95% confidence interval) is based on the assumed risk in the comparison group and the relative effect of the intervention (and its 95% CI).

CI: confidence interval; RR: risk ratio.

^a^ Serious concerns for risk of bias (minus one grade).

^b^ Serious concerns for indirectness due to study population being limited to sub-population of migrant workers (minus one grade).

^c^ Very serious concerns for risk of bias (minus two grades).

^d^ Very serious concerns for imprecision indicated by the 95% confidence estimate or estimates ranging from a meaningful benefit to a meaningful harm (minus two grades).

The body of evidence synthesised in this systematic review and meta-analyses has several limitations. Clinical heterogeneity was high across populations and some outcomes. Migrant workers as a population are diverse in terms of country of origin, stage of migration, formality of migration (regular versus irregular), country of destination, occupation, industrial sector, work in the formal versus informal economy, lengths of residence in the country of destination, gender composition, and level of education, amongst other variables. Differences in health service use and health outcomes are also influenced by up-stream, structural interventions, such as whether migrant workers are covered with employment injury compensation schemes. These upstream interventions will differ between countries. The high clinical heterogeneity observed in this systematic review may reflect these differences in migrant populations and work-related policies and programmes in the studied countries. Some outcomes were also relatively heterogenous in one or both of definition and measurement. Statistical heterogeneity was also high in the meta-analyses for most outcomes (I^2^ ≥ 95%). We caution the interpretation of the pooled estimates and suggest these are viewed as indicative, not conclusive.

We judged the current body of evidence to be seriously indirect for most outcomes included in this review. The existing evidence covers a subpopulation of migrant workers. These are primarily migrant workers at the country of destination, residing in high-income destination countries, who have migrated regularly and work in the formal economy. Some of the migrants covered in the included studies are North-North migrants. There is scarce scientific evidence is available on migrant workers coming from low income country and migrating to low-middle income countries. Therefore, this current body of evidence does not capture migrant workers in more vulnerable situations, including those in transit (e.g., in detention) and return (e.g., those who returned after acquiring an occupational or work-related injury or disease in the destination country); migrant workers residing in low- and lower middle-income countries; those who have migrated irregularly (and therefore do not enter civil registration and in turn may not qualify for public services and benefits); and migrants working in informal economies. The evidence reviewed can, therefore, only partially answer the systematic review’s research questions.

High risk of bias presents a serious concern in this systematic review for several outcomes. Most included studies investigate non-representative samples. Even probability studies or complete censuses of general populations (e.g. all occupational injuries registered in the national injury register or employment injury compensation scheme) have generally either actively or *de facto* excluded irregular migrant workers because their lack of civil registration and entitlement for compensation means that they do not appear in official registries and compensation records, respectively. There is a high risk of bias from disproportionate underreporting of cases of occupational and work-related diseases or injuries due to fear of losing one’s job among migrant workers. For this reason, different approaches to data collection may be necessary in future research: approaches need to be able to capture information on the health outcomes of all migrant workers, including those in the informal economy or with irregular status.

The body of evidence is limited to comparisons of migrant workers in destination countries with non-migrant workers in the same country. If migrant workers who experience changes in health services or health outcomes return to their country of origin as a result (e.g., because they lose their right of residency as soon as they are no longer fit for work or have lost their employment), they are lost from official registries and national cohort studies. The adverse health effects they experienced will also not be captured in these national data and information systems. This can lead to an underestimation of the RR of migrant workers compared with non-migrant workers. Studies that compare migrant workers pre-departure in their country of origin with the same migrant workers after return to their country of origin are needed. These studied were eligible for inclusion in this systematic review, but we did not identify any.

This review was limited to migrant workers who had entered their country of destination through regular migration (with few exceptions). Fitness to work established through medical examination is often a prerequisite for regular migration for employment. This would lead to selection based on health status, where applicants for migration who are in less than ideal health are excluded from regular migration, and only relatively healthy persons can enter a country of destination as regular migrant workers. Additionally, regular migration often also depends on the applicant fulfilling minimum education and wealth requirements that generally lie above the average level achieved in non-migrant workers in the country. In summary, this health selection may explain any health advantage observed in regular migrant workers, compared with a general sample of non-migrant workers in the same country.

This review highlights that more high-quality research is needed on these differences in workers’ health services use and health outcomes by migrant status. More research is required on broader and more diverse populations of migrant workers, particularly on the forms of migration and the stages of migration, in which more careful identification of migrant workers and measurement of their health status is made. Longitudinal studies, such as cohort studies, may be particularly informative; however, large scale population-based studies, using data from population censuses for example, are also needed. This could include research on migrant workers from and in low-income countries, in migration stages other than destination, from irregular migration, and migrants working in the informal economy. Harmonized internationally standardized measures will aid comparability between studies. Further research will strengthen the evidence base for designing, planning, costing, implementing and evaluating laws, policies, and interventions that promote the safety, health and wellbeing of workers.

## Supporting information

S1 Checklist PRISMA 2009 checklist. (DOC)

S1 Appendix Search strategy for MEDLINE. List of search terms used in MEDLINE to identify literature for inclusion in the systematic review.
(DOCX)

S1 Table Eligibility criteria. (DOCX)

S2 Table Prioritized outcomes and related Sustainable Development Goals indicators. (DOCX)

S1 Fig Summary of risk of bias. Figure summarising the risk of bias for included studies.
(DOCX)

S2 Fig Risk of bias graph. Figure summarising the risk of bias for included studies.
(DOCX)

S3 Fig Has had any occupational injury, migrant workers compared with non-migrant workers, 2010–20, sensitivity analysis. Forest plot of sensitivity analysis for outcome of “Had any occupational injury” with the López-Arquillos 2016 study included using the median standard error across the included individual effect estimates from the other included studies (0.08).
(DOCX)

S4 Fig Has HIV, migrant workers compared with non-migrant workers, 2010–20. Figure showing the odds ratios (and 95% confidence intervals) of reported in included studies of having HIV among migrant workers compared with non-migrant workers, 2010–2020.
(DOCX)

S5 Fig Is clinically depressed, migrant workers compared with non-migrant workers, 2010–20. Figure showing the odds ratios (and 95% confidence intervals) of reported in included studies of being clinically depressed among migrant workers compared with non-migrant workers, 2010–2020.
(DOCX)
