# Supplementary material for: Health service use and health outcomes among international migrant workers compared with non-migrant workers: A systematic review and meta-analysis
Source: PLoS One. 2021 Jun 9;16(6):e0252651. doi: 10.1371/journal.pone.0252651 (PMC8189512; doi:10.1371/journal.pone.0252651)
Supplement: S1 Appendix — List of search terms used in MEDLINE to identify literature for inclusion in the systematic review. (DOCX) [file pone.0252651.s002.docx]

### **S1 Appendix: Search strategy for MEDLINE**

1 Refugees/

2 "Transients and Migrants"/

3 "Undocumented Immigrants"/

4 "Emigrants and Immigrants"/

5 "Emigration and Immigration"/

6 Human Trafficking/

7 or/1-6

8 exp Work/

9 exp Occupational Groups/

10 Sex Workers/

11 Working Poor/

12 exp Employment/

13. or/8-12

14. 7 and 13

15 ((refugee* or migrant* or immigrant* or emigrant* or stateless* or foreign* or asylum* or noncitizen* or displaced or seasonal* or circular* or cyclical*) adj3 (work* or labor* or labour* or employ*)).ti,ab.

16 (((new* or recent*) adj3 (arrival* or arrived)) and (work* or labor* or labour* or employ*)).ti,ab.

17 ((human or sex) adj3 traffick*).ti,ab.

18 or/15-17

19 14 or 18

20 exp Community Health Nursing/

21 Community Mental Health Services/

22 exp Counseling/

23 Family Planning Services/

24 Health Services for Transgender Persons/

25 exp Mental Health Services/

26 exp Preventive Health Services/

27 exp Reproductive Health Services/

28 exp General Practice/

29 exp Primary Health Care/

30 or/20-29

31 19 and 30

32 Occupational Health Services/

33 exp Rehabilitation,Vocational/

34 ((occupational* or workplace* or industrial* or vocational*) adj3 (health* and service*)).ti,ab.

35 or/32-34

36 19 and 35

37 Occupational Health/

38 exp Occupational Diseases/

39 Occupational Injuries/

40 Accidents, Occupational/

41 Workplace Violence/

42 ((occupational* or work* or labor* or labour* or employ*) adj3 (death* or died or fatal* or mortalit* or disease* or illness* or injur* or accident*)).ti,ab.

43 or/37-42

44 19 and 43

45 Mental Health/

46 exp Depressive Disorder/

47 exp "Suicide"/

48 (depress* or suicide*).ti,ab.

49 or/45-48

50 19 and 49

51 exp HIV Infections/

52 exp HIV/

53 Acquired Immunodeficiency Syndrome/

54 HIV infection.mp.

55 human immunodeficiency virus.mp.

56 (acquired immun* and deficiency syndrome).mp.

57 AIDS.ti,ab.

58 or/51-57

59 19 and 58

60 31 or 36 or 44 or 50 or 59

61 limit 60 to yr="2010 -Current"
