# Supplementary material for: Health service use and health outcomes among international migrant workers compared with non-migrant workers: A systematic review and meta-analysis
Source: PLoS One. 2021 Jun 9;16(6):e0252651. doi: 10.1371/journal.pone.0252651 (PMC8189512; doi:10.1371/journal.pone.0252651)
Supplement: S1 Table — (DOCX) [file pone.0252651.s003.docx]

### **S1 Table: Eligibility criteria**

| **Domain** | **Eligibility criterion** |
| --- | --- |
| Types of studies | Included:   - Studies with records published between Jan 1, 2010, and Feb 29, 2020. - Study records written in any language. - Studies of any quantitative design, including cross-sectional studies. - Studies providing estimates of relative differences between migrant and non-migrant workers in an eligible outcome, such as a risk, hazard or odds ratio.   Excluded:   - Qualitative studies, case reports, and modelling studies. - Study records without quantitative data (e.g., letters, commentaries and perspectives). - Studies with estimates of absolute differences only (e.g. difference in means or risks). - Studies that reported the prevalence or the incidence for eligible migrant workers and eligible non-migrant workers but did not report an estimate of the relative differences.   If a study presented models for different migrant populations (e.g., Model A for migrant workers who had ever arrived in the study country, Model B for migrant workers who had arrived in the last 6 years, and Model C for migrants who had arrived in the last 3 years), we prioritised the most inclusive model (i.e., in our example: Model A). We prioritised confounder-adjusted models over crude models and prioritised the model that we considered best adjusted for confounding (e.g., prioritised a model adjusted for two potential confounders over a model adjusted for one of the two confounders only). If a study presented two or more models adjusted for exclusive sets of confounders, we prioritised the model that we considered most relevant (e.g., prioritised a model adjusted for type of industry only over a model adjusted for age only). If a study presented data for multiple years, we prioritised the latest data for inclusion in the review. |
| Types of populations | Included:   - International migrant workers, as defined by ILO.^1^ - Trafficked workers. - Migrant workers who were a refugee or seeking asylum, including internationally displaced people who work. - Country of birth, ethnicity or nationality indicative of migration (e.g., foreign-born Tunisians or nationals of China working in India). - Workers who had migrated regularly (e.g., through official channels) and those who had migrated irregularly (e.g., not through official channels, such as undocumented migrants). - Migrant workers in the formal economy and those in the informal economy. - Temporary, short-term and long-term migrant workers, including permanent, seasonal and circular or cyclical ones. - Working-age adults (defined as 15-64 years). - Migrant workers at any of the five stages of migration (i.e., pre-departure, travel, destination, intersection, and remigration stage).^2^ - Any geographic setting and unit. - Workers in any economic sector or occupational group, including health and social care workers.   Excluded:   - Internal (within-country) migrant workers. - Forcibly displaced workers who had not crossed an international border. - Migrants who were not explicitly identified as workers (e.g., studies of all working-age migrants, including those not working or seeking work). - Families of migrant workers, whether left behind in the country of origin or accompanying the migrant worker in the destination country. - Indigenous ethnic groups who live and work nomadically, even if their work crossed borders. - Ethnicity or nationality not based on migration status (e.g., person of Latino ethnicity residing in Italy). - Children (≤15 years) and older adults (≥65 years). |
| Types of comparator | Included: Non-migrant workers (e.g. non-migrant workers in the country of destination and in the migrant workers’ country of origin, whether before departure from the country of origin or after return to it).  Excluded:   - All other comparators (e.g. migrant workers from another country of origin and not exclusively migrant workers (e.g., only 90% were international migrants). - Studies using the total population as the comparator. |
| Types of outcomes | Included:   - Has used any health service. - Has used any occupational safety and health service. - Has died from an occupational injury. - Has had any non-fatal occupational injury. - Has human immunodeficiency virus infection. - Has clinical depression.   Excluded: All other outcomes. |

References

1. International Labour Organization. Decent work for migrants and refugees. Geneva: International Labour Organization. Available from: <http://www.ilo.org/wcmsp5/groups/public/---dgreports/---dcomm/documents/publication/wcms_524995.pdf>; 2016.

2. Zimmerman C, Kiss L, Hossain M. Migration and health: a framework for 21st century policy-making. *PLoS medicine* 2011; **8**(5): e1001034.
