# Supplementary material for: Health service use and health outcomes among international migrant workers compared with non-migrant workers: A systematic review and meta-analysis
Source: PLoS One. 2021 Jun 9;16(6):e0252651. doi: 10.1371/journal.pone.0252651 (PMC8189512; doi:10.1371/journal.pone.0252651)
Supplement: S2 Table — (DOCX) [file pone.0252651.s004.docx]

### **S2 Table: Prioritized outcomes and related Sustainable Development Goals indicators**

|  | **Outcome in this review** | **Relevant Sustainable Development Goals indicator** |
| --- | --- | --- |
| 1 | Has used any health service | 3.8.1 Coverage of essential health services (defined as the average coverage of essential services based on tracer interventions that include reproductive, maternal, newborn and child health, infectious diseases, non-communicable diseases and service capacity and access, among the general and the most disadvantaged population) |
| 2 | Has used any occupational safety and health service |  |
| 3 | Has died from an occupational injury | 8.8.1 Frequency rates of fatal and non-fatal occupational injuries, by sex and migrant status |
| 4 | Has had any non-fatal occupational injury |  |
| 5 | Has human immunodeficiency virus infection | 3.3.1 Number of new HIV infections per 1,000 uninfected population, by sex, age and key populations |
| 6 | Has clinical depression | 3.4.2 Suicide mortality rate |
