# Supplementary figures and images for: Health service use and health outcomes among international migrant workers compared with non-migrant workers: A systematic review and meta-analysis
Source: PLoS One. 2021 Jun 9;16(6):e0252651. doi: 10.1371/journal.pone.0252651 (PMC8189512; doi:10.1371/journal.pone.0252651)

### **S1 Fig:** Summary of risk of bias

**
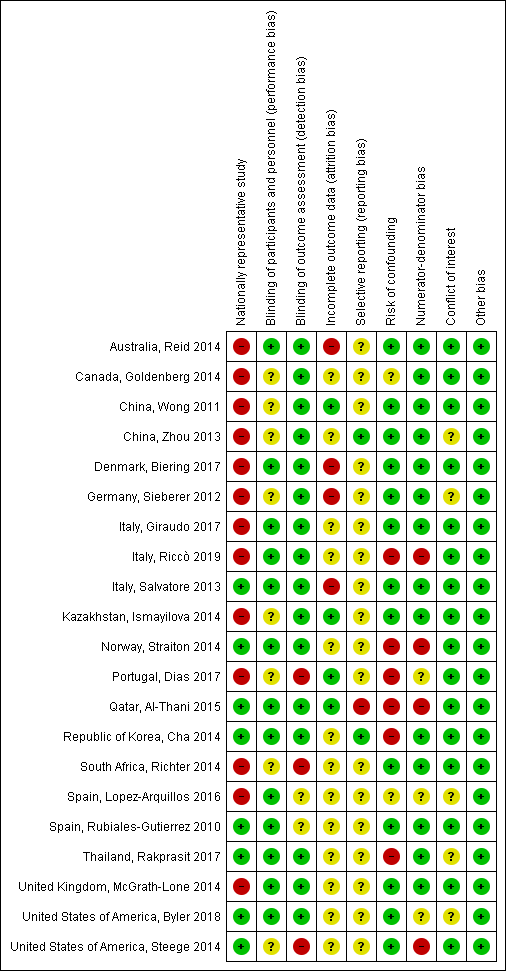
**

Supplement: S1 Fig — Figure summarising the risk of bias for included studies. (DOCX) [file pone.0252651.s005.docx]

### **S2 Fig: Risk of bias graph**


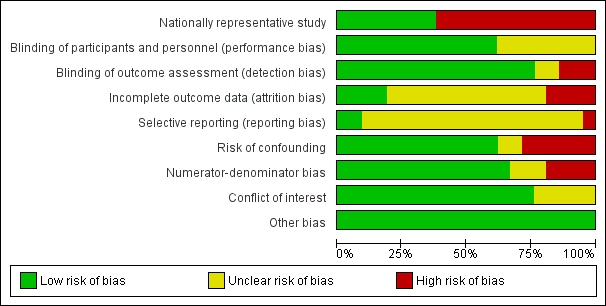

Supplement: S2 Fig — Figure summarising the risk of bias for included studies. (DOCX) [file pone.0252651.s006.docx]

### **S4 Fig:** Has HIV, migrant workers compared with non-migrant workers, 2010-20

**
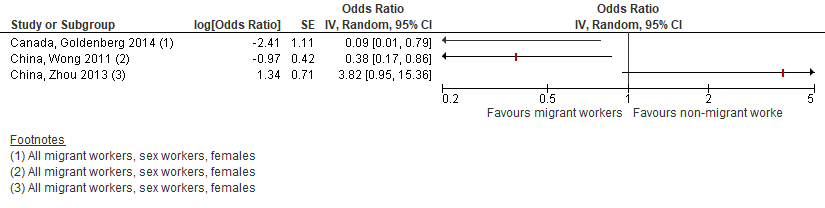
**

Supplement: S4 Fig — Figure showing the odds ratios (and 95% confidence intervals) of reported in included studies of having HIV among migrant workers compared with non-migrant workers, 2010–2020. (DOCX) [file pone.0252651.s008.docx]

### **S5 Fig:** Is clinically depressed, migrant workers compared with non-migrant workers, 2010-20

**
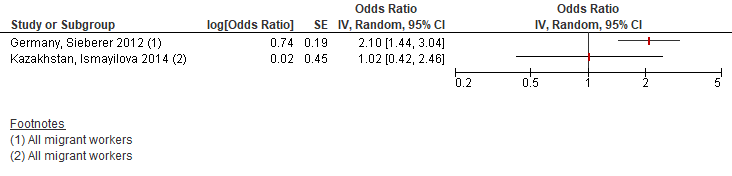
**

Supplement: S5 Fig — Figure showing the odds ratios (and 95% confidence intervals) of reported in included studies of being clinically depressed among migrant workers compared with non-migrant workers, 2010–2020. (DOCX) [file pone.0252651.s009.docx]
