# Supplementary material for: Health service use and health outcomes among international migrant workers compared with non-migrant workers: A systematic review and meta-analysis
Source: PLoS One. 2021 Jun 9;16(6):e0252651. doi: 10.1371/journal.pone.0252651 (PMC8189512; doi:10.1371/journal.pone.0252651)

### **S3 Fig: Has had any occupational injury, migrant workers compared with non-migrant workers, 2010-20, sensitivity analysis**

Forest plot of sensitivity analysis for outcome of “Had any occupational injury” with the López-Arquillos 2016 study included using the median standard error across the included individual effect estimates from the other included studies (0.08)


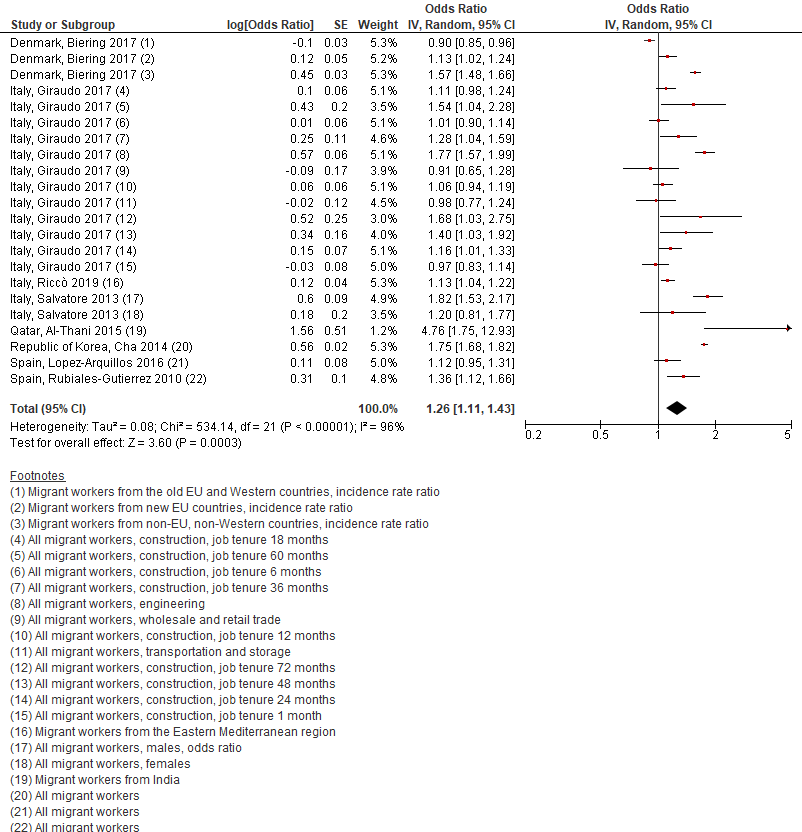

Supplement: S3 Fig — Forest plot of sensitivity analysis for outcome of “Had any occupational injury” with the López-Arquillos 2016 study included using the median standard error across the included individual effect estimates from the other included studies (0.08). (DOCX) [file pone.0252651.s007.docx]
